# Output-Based Structural Damage Detection by Using Correlation Analysis Together with Transmissibility

**DOI:** 10.3390/ma10080866

**Published:** 2017-07-27

**Authors:** Yun-Lai Zhou, Hongyou Cao, Quanmin Liu, Magd Abdel Wahab

**Affiliations:** 1 Department of Civil and Environmental Engineering, National University of Singapore, Singapore 117576, Singapore; ceezyl@nus.edu.sg; 2 School of Civil Engineering and Architecture, Wuhan University of Technology, Wuhan 430070, China; 3 Engineering Research Center of Railway Environmental Vibration and Noise of the Ministry of Education, East China Jiaotong University, Nanchang 330013, China; qmlau007@126.com; 4 Institute of Research and Development, Duy Tan University, 03 Quang Trung, Da Nang 550000, Vietnam; magd.abdelwahab@UGent.be; 5 Soete Laboratory, Faculty of Engineering and Architecture, Ghent University, Technologiepark Zwijnaarde 903, B-9052 Zwijnaarde, Belgium

**Keywords:** damage detection, transmissibility, dynamic analysis, correlation analysis

## Abstract

Output-based structural damage detection is becoming increasingly appealing due to its potential in real engineering applications without any restriction regarding excitation measurements. A new transmissibility-based damage detection approach is presented in this study by combining transmissibility with correlation analysis in order to strengthen its performance in discriminating damaged from undamaged scenarios. From this perspective, damage detection strategies are hereafter established by constructing damage-sensitive indicators from a derived transmissibility. A cantilever beam is numerically analyzed to verify the feasibility of the proposed damage detection procedure, and an ASCE (American Society of Civil Engineers) benchmark is henceforth used in the validation for its application in engineering structures. The results of both studies reveal a good performance of the proposed methodology in identifying damaged states from intact states. The comparison between the proposed indicator and the existing indicator also affirms its applicability in damage detection, which might be adopted in further structural health monitoring systems as a discrimination criterion. This study contributed an alternative criterion for transmissibility-based damage detection in addition to the conventional ones.

## 1. Introduction

Structural health monitoring (SHM) plays an essential role in many different fields, such as civil engineering and mechanical engineering; particularly in aerospace engineering, SHM performs as an indispensable task in guaranteeing the integrity of entire structures such as aeroplanes, aircraft, and so on, due to the high integration of advanced materials and complex connections in these structures, which usually are subjected to complex loadings. Since aerospace structures contain complex materials and sophisticated connections, the inspection technique for detecting defects can be summarized into several different categories: (1) metal (steel/aluminum and so on) structure-based SHM; (2) composite structure-based SHM; (3) electrical system-based SHM; and (4) others, such as plastic structure-based SHM. Usually, the metal and composite structure-based SHM are considered together as mechanical failure warnings. Over the last decades, a large amount of techniques have been raised and developed to identify damages in these kinds of structures, in order to provide early-stage warnings for potential maintenance and repairing. The most common technique would be vibration-based techniques, which rely on modal testing and further data analysis. For instance, in ref. [1], the subspace-based modal analysis method is introduced into damage detection for on-line monitoring under an unknown excitation applied to a Paris MS760 airplane. The damage is modeled by changing the mass and stiffness in the airplane structure. The adoption of this vibration-based technique relies on its cost-effective performance in damage identification. The kernel behind this is that damage often results in the change of physical parameters such as stiffness, which will further affect the structural dynamic responses. For a critical and thorough review of vibration-based techniques, please see ref. [2]. In ref. [3], SHM for aerospace structures is systematically reviewed, summarizing the technical evolution history and illustrating the technology from two perspectives; i.e., measuring techniques and the evolution of methodologies. For measuring techniques, new sensors like fiber bragg grating (FBG), non-destructive testing such as acoustic emission (AE), radiography, thermography and so on are systematically introduced; as for technical evolution, signal processing techniques like filters, artificial neurons, wavelet transforms and so on are generally described.

For the health monitoring of aerospace structures, the aerospace structure is usually considered in components rather than the entire system; for each component, a specific technique is applied to inspect and monitor the state during its aging cycle. For instance, in Ref. [4], AE, optical and strain gaging methods are introduced for detecting flaws; the AE allowing the detection of growing flaws at 10% load, but the optical method failing to detect a 1 mm fatigue crack in wing section. AE also allows the detection of invisible damage in aircraft composite panels [5]. In addition, visual inspection such as automated exterior inspection with a mounted camera is also utilized [6], while other methods like lamb wave can certainly also detect the damages in aircrafts; e.g., damage in the aircraft wing [7].

Even if new techniques such as AE, ultrasonic testing and eddy currents are raised for detecting damage, it would be time-consuming and challenging in some large scale component testing. For a global scale structural inspection, vibration-based techniques still perform as the most cost-effective option, especially after the great improvement over the last decades. Techniques are raised in the time-domain and frequency domain, with both empirical and model-based approaches [2]. Conventional modal parameters such as natural frequencies, frequency response functions (FRFs), mode shape and its derivatives are frequently extracted from experimental modal analysis (EMA), in which the measurement of excitation restricts its further application in real engineering structures. In order to solve this problem, output-based techniques are thus developed; so-called operational modal analysis (OMA). Among all vibration-based techniques, transmissibility occupies a significant role as it only relies on the structural responses, without any requirement on the excitation measurement [8,9,10,11,12,13,14,15]. Transmissibility has been shown to perform well in damage detection, and is applied to damage detection [8,9,12,13,14], localization [10], and quantification [10,11,15]. Transmissibility is critically reviewed, recalling the technical evolution history and engineering application [10]. Transmissibility, a concept defined in [16,17], can be used to characterize structural dynamic properties; for instance, to extract natural frequencies. The interrelation between transmissibility and structural physical properties shows the potential in characterizing the structural state; i.e., identifying structural damages. For instance, in ref. [8], principal component analysis (PCA) is applied to reduce the dimension of transmissibility, then the distance measure is applied to enhance damage detection performance; in ref. [9], transmissibility works together with Mahalanobis distance and Hotelling T square for damage detection; in [11], a new concept—transmissibility coherence—is raised and applied to damage detection and quantification; transmissibility can also work with distance measuring [12], clustering analysis [13], neural networks [14], and similarity measuring [15] in identifying the structural damages.

In SHM, the damage state of a system can be described as a five-step process along the lines of the process discussed in Rytter (1993) [16]. The damage state is described by answering the following questions [2]: (1) Is there damage in the system (existence)? (2) Where is the damage in the system (location)? (3) What kind of damage is present (type)? (4) How severe is the damage (extent)? (5) How much useful life remains (prognosis)? In [2], the SHM problem is described as one of a statistical pattern recognition paradigm, which consists of a four-part process: (1) Operational Evaluation; (2) Data Acquisition, Fusion, and Cleansing; (3) Feature Extraction and Information Condensation; (4) Statistical Mode Development for Feature Discrimination. This provides an advantage compared to conventional methodologies, as this four-part process only relies on the structural measured responses and does not demand any physical properties in advance, which gives a high probability for its application in real engineering. It may be necessary to offer more detail concerning damage, which is defined as changes introduced to a system which adversely affect its current or future performance [2]. From this respect, damage would involve various kinds of structural performances, such as surface cracking [17,18,19], internal defects [20], debonding in composites, spalling in concrete structures, and so on. Certainly, it is also possible to consider factors such as corrosion, fatigue loading [21], and so on as damage. It is also feasible to consider the consequences of the damage, such as stiffness change [8,9,10,11,12,13,14,15] or damping change [22]. Accordingly, SHM methodologies can be chosen from different perspectives. The reasoning behind this is that a kind of damage can be successfully detected from one aspect while it would be arduous or even unsuccessful when taking another perspective. For instance, small spalling in concrete structures may not result in global stiffness or damping change. Therefore, if taking the consequences into consideration when designing SHM systems, this would be challenging, while it would be much more easier to design a SHM system if using image recognition [23]. In addition, another interesting issue is the nonlinearity problem, including material nonlinearity, geometric nonlinearity and boundary conditions, within which hysteretic damping [22] is a research focus, which seems to enable stiffness-based damage detection to become challenging. However, it would obtain the opposite conclusion if one directly employed damping-sensitive measured data to characterize the current damping. The kernel in SHM is to focus on the main factor, while it might be not of the same necessity and importance to take the less essential factors into consideration [2]. In summary, three perspectives—(1) damage performance; (2) damage cause; (3) damage correspondence—should be taken into consideration in the initial stage of SHM; how to select the perspective depends on the engineers’ experience and the available sources.

Whichever kind of approaches have been developed, early-stage damage-detection still presents challenges, and drawing out a strong feature insight to detect damage is still pursuable and indispensable from a point of view. In real engineering, environmental variety also influences the further detection of damages, meaning that it is not easy to detect structural damages in a completely successful manner. Hence, this opens the possibility to figure out more techniques to detect damage efficiently and effectively. Previous studies in SHM have certainly already provided some, or even various, methodologies to identify damages, while obstacles still remain, especially in highly integrated structures such as aerospace craft. The interference between components makes SHM still more arduous. For instance, the online SHM for rotor blades in turbine engine is still awaiting proposals, where the high-temperature, high-speed operating rotor blade will also induce interference to its adjacent components’ online SHM, such as defects in the rotor blade or the fretting wear in the rear holder.

In this study, a new transmissibility-based damage detection approach using correlation analysis is proposed. Considering a vibrated elastic structural system, transmissibility can be extracted directly from the vibration response without considering the excitation. As transmissibility holds a sensitive interrelation with the structural characteristics, i.e., dynamic damage, damage-sensitive indicators can be defined using structural transmissibility. The correlation analysis for transmissibility opens the possibility to construct an easier but effective indicator. For comparison reasons, the correlation-based damage indicator is compared with a previously investigated damage indicator. For methodology testing and validation, a cantilever beam is numerically analyzed, and an ASCE benchmark is also taken into consideration to evaluate the feasibility of the proposed technique. The main contribution of this study is to establish damage-sensitive indicators considering the correlation perspective, and gives a potential application in engineering damage-detection in addition to the conventional approaches.

## 2. Theoretical Background

### 2.1. Transmissibility

As defined in [24,25], for a harmonic applied force at a given coordinate, the transmissibility of an elastic system between point *i* and *p* can be defined as:

(1) $$T_{\left(i,p\right)}(\omega )=\frac{X_{i}(\omega )}{X_{p}(\omega )}$$

where *X_i_* and *X_p_* are the complex amplitudes of the system responses *x_i_*(*t*) and *x_p_*(*t*), respectively. Previous investigations have proven its feasibility and capacity in damage identification [26,27], and a recent summary can be found in [28].

In real engineering or experiment analysis, many kinds of approaches can be utilized to extract the system transmissibility; for example, using FRFs:

(2) $$T_{\left(i,p\right)}(\omega )=\begin{array}{c} X_{i}(\omega )/F_{b}(\omega )\\ X_{p}(\omega )/F_{b}(\omega ) \end{array}=\begin{array}{c} H_{i,b}(\omega )\\ H_{p,b}(\omega ) \end{array}$$

where *b* is the excitation point, and *H* represents the FRF.

In addition, auto-spectrum and cross-spectrum can also be taken to estimate the transmissibility; one possible description can be expressed as:

(3) $$T_{\left(i,p\right)}(\omega )=\begin{array}{c} X_{i}(\omega )\times X_{p}(\omega )\\ X_{p}(\omega )\times X_{p}(\omega ) \end{array}=\begin{array}{c} G_{i,p}(\omega )\\ G_{p,p}(\omega ) \end{array}$$

where *G* is the auto or cross-spectrum. From this point, one may extract transmissibility coherence (TC), and for detailed information, the reader may refer to [10,11]. In this study, transmissibility is estimated by Equation (3). Certainly, one may estimate the transmissibility via other approaches, but the results would be identical.

Certainly, Equations (1)–(3), especially Equations (2) and (3), serve for single input single output (SISO) or single input multiple output (SIMO) linear systems, while it would be a bit complex for a multiple input multiple output (MIMO) system. The definition still follows with the form and kernel of Equation (1), while the derivation would desire some further extension in comparison with Equations (2) and (3). Several attempts have even been made, but mainly methodologies are developed in two direction; i.e., forward [24,25] and backward [29]. These definitions have been applied in FRF evaluation [30] and OMA [31,32]. The forward method [24,25] tries to illustrate the transmissibility in a MIMO system, which mainly functions to estimate the unknown responses from the known ones, while the backward way [29] intends to identify the system parameters from the derived transmissibilities, which relies on the fact that the peaks of the delta inverse transmissibilities coincide with the natural frequencies [31].

Another issue here that might be of interest is the reference selection; in terms of this issue, previous investigations have seldom discussed it. It is claimed that this selection depends on engineering experience [10], and further, some suggestions and basic rules are raised in [8]; the key idea addressed is that the transmissibility would occupy more storage space and further processing capacity, so that the selection of its characteristic part in light of a structural system would typically represent the structural state; for example, one column of the whole transmissibility matrix. Certainly, in addition to the artificial selection, it would also be feasible to use some algorithm such as PCA [8]. In this study, the transmissibility selection relied on the engineers’ experience, which will be further addressed in detail in the analysis of the results.

### 2.2. Damage Sensitive Feature

Correlation has been a widely-studied approach for detecting damage, although most studies concentrate on the cross correlation between two signals. In this study, we have tried to estimate the correlation between the transmissibility under testing conditions and the baseline, i.e., the transmissibility under intact condition using a Pearson correlation coefficient.

Pearson correlation coefficient, raised by Karl Pearson (1880s), is a measure to estimate the correlation between two datasets. It has been used to detect damage; for instance, in [33], the Pearson correlation coefficient is utilized to assess the correlation between the waveform with the baseline, and thus to construct the damage indicator signal difference coefficient (SDC), which has been successfully used in multiple challenging damage detection specimens [34,35]. In addition, in [34], SDC has been applied to damage detection in aircraft wings, where the complicated shape normally makes it challenging to identify damages; in [35], SDC is incorporated with tomography algorithms in detecting damages in aluminum and composite plate. Pearson correlation coefficient is defined as:

(4) $$Cor(Y,Z)=\frac{Cov(Y,Z)}{\sigma_{Y}\sigma_{Z}}=\frac{\sum_{t=1}^{n}\left(Y_{t}-\bar{Y})(Z_{t}-\bar{Z}\right)}{\sqrt{\sum_{t=1}^{n}{\left(Y_{t}-\bar{Y}\right)}^{2}}\sqrt{\sum_{t=1}^{n}{\left(Z_{t}-\bar{Z}\right)}^{2}}}$$

where *Y*, *Z* mean two datasets with their average $\bar{Y}$, $\bar{Z}$, *t* in *Z_t_* means the variable number from 1 to n, and *n* means total number of variables, *Cov* means the covariance and σ means the product of the standard deviations.

From the Pearson correlation coefficient shown above, the correlation-based damage indicator (*CDI*) can be defined as:

(5) $$CDI(T^{d},T^{u})=abs(\frac{Cov(T^{d},T^{u})}{\sigma_{T^{d}}\sigma_{T^{u}}})=abs\left(\frac{\sum_{t=1}^{n}\left(T_{t}^{d}-{\bar{T}}^{d})(T_{t}^{u}-{\bar{T}}^{u}\right)}{\sqrt{\sum_{t=1}^{n}{\left(T_{t}^{d}-{\bar{T}}^{d}\right)}^{2}}\sqrt{\sum_{t=1}^{n}{\left(T_{t}^{u}-{\bar{T}}^{u}\right)}^{2}}}\right)$$

where *T^d^*, *T^u^* mean the transmissibility under damaged condition and undamaged condition, respectively. Note that in this case, *CDI* ∈ [0, 1], and when *CDI* approaches ‘1’, it means no damage occurrence; if *CDI* approaches ‘0’, it means that damage is confidently occurred.

In addition to *CDI*, for comparison reason, since transmissibility is a vector for each structural state, then modal assurance criterion (MAC) can be utilized to generate a damage-sensitive indicator, transmissibility assurance criterion (*TAC*), which can be indicated as:

(6) $$TAC=\frac{{\left({\left(T_{}^{d}\right)}^{T}(T_{}^{u})\right)}^{2}}{\left({\left(T_{}^{d}\right)}^{T}(T_{}^{d})\right)\left({\left(T_{}^{u}\right)}^{T}(T_{}^{u})\right)}$$

where *T^d^* means transmissibility under a damaged condition, while *T^u^* means the transmissibility under intact condition, ()*^T^* means the transpose of (). As discussed in ref. [10], for *TAC*, the frequency span chosen is important; this indicator *TAC* is not for the transmissibility in whole frequency span, which means that one should select a frequency span for the indicator, as some part of the frequency span might be contaminated by the environmental noise. Note also that *TAC* ∈ [0, 1] and that when *TAC* approaches ‘1’ it means no damage occurrence; if *TAC* approaches ‘0’, it means that damage is confidently occurred. Thus, *TAC* can be compared with *CDI* discussed above, and these two indicators will allow the determining of the capacity in inspecting damages.

A recent investigation unveiled the hidden reason between MAC and angle estimator—cosine similarity measure [15]—where it gives the hidden reason why MAC can be applied to optimization analysis and can be utilized for the construction of objective functions. The reason is that the MAC aims to estimate the square of the cosine measure of the angle between two vectors. In this study, the use of *CDI* and *TAC* is in order to unveil the feasibility in damage inspection for SHM. Note, a threshold should be set for the indicators, and the value under the intact condition is set as a threshold in this study.

### 2.3. Damage Detection Procedure

**Figure 1:**
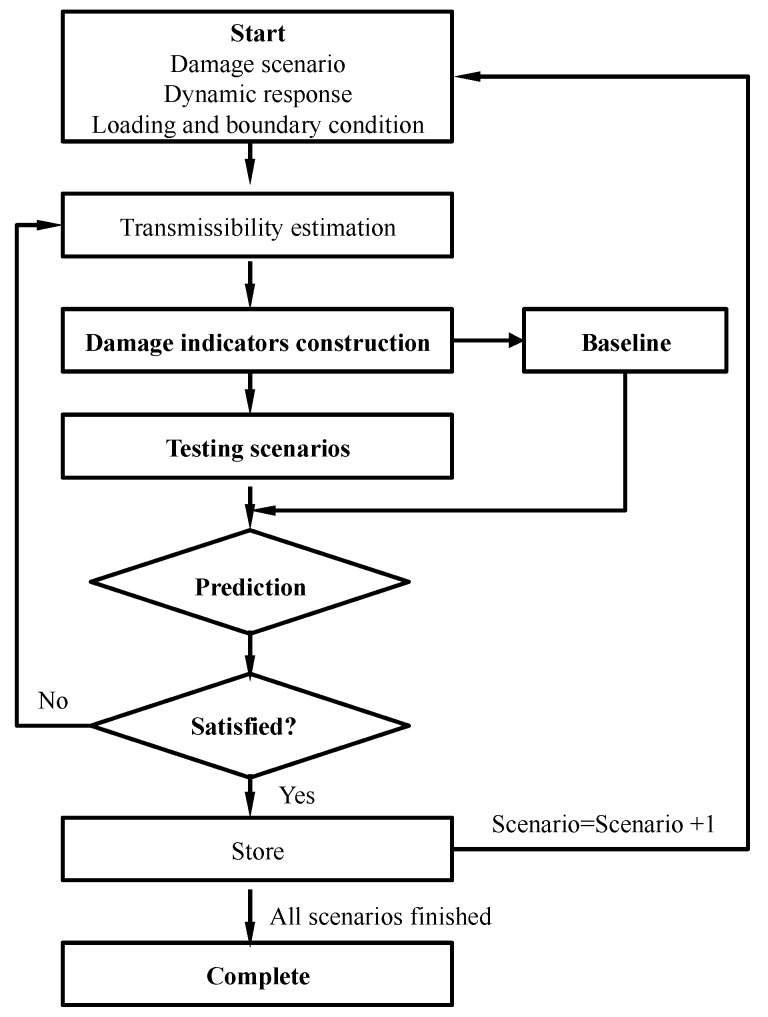
Flowchart of the transmissibility-based damage detection procedure.

In order to draw out the clear procedure for the proposed damage detection methodology, this section tries to explain the procedure for detection damage in detail as follows, with a clear framework shown in Figure 1:

***Step 1:*** *Transmissibility estimation.* Transmissibility is assessed from the structural dynamic responses with Equation (3) for all scenarios, including undamaged and damaged scenarios. Certainly, one may also use Equation (2) here to assess the transmissibility; the results will be consistent. In terms of transmissibility selection, this study follows the suggestions raised in ref. [8], and selected columns are considered in further analysis;

***Step 2:*** *Damage indicator derivation.* The transmissibility-based damage indicators are derived using Equations (5) and (6) for each scenario, which will be hereafter taken into the damage prediction process. Note that the baseline means the undamaged scenario, which is set as the intact pattern to be compared with in the further analysis as shown in Figure 1, and this baseline can be constructed from averaging a certain amount of measurements under the undamaged scenario; 

***Step 3:*** *Damage prediction.* According to the threshold set before, the damage will be predicted and compared with the intact pattern; herein, the threshold set is also an essential issue; certainly, this can also rely on the engineers’ experience, since no fixed criteria can adapt to all the environmental circumstances; the kernel is that the difference between the intact pattern and damaged scenarios should be clear and can be visualized; 

***Step 4:*** *Result confirmation and storage.* This step might be essential in a damage detection procedure, as a false alarm might be encountered. Then, it is indispensable to confirm the predicted results and store those confirmed results. As shown in Figure 1, this step includes checking whether the predicted results are satisfied or not, if the results are satisfied according to the engineers’ experience; the predictions would then be stored and the next scenario started. If not, it would be necessary to go back to the transmissibility estimation, and restart the detection procedure again.

## 3. Numerical Analysis

### 3.1. Model Description 

**Figure 2:**
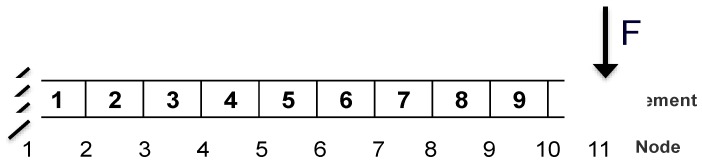
A schematic diagram of the cantilever beam.

In order to check the feasibility of the developed damage detection procedure, a cantilever beam with ten elements is numerically analyzed in this section. As shown in Figure 2, the dimensions of the beam are 500 × 50 × 6 mm^3^ in length, height, and thickness, respectively. Young’s modulus is 185.2 GPa and the density is 7800 kg/m^3^. A slight damping is considered with damping ratio 0.02.

An impulse with maximum amplitude 1 N is applied at node 11, and damage is modeled with stiffness loss. As discussed in [36], it is possible to model structural damage with stiffness loss corresponding to the dimensional change, such as a saw cut in a beam. Note that in this case, other kinds of damage without modifying the structural stiffness are not considered. Hereafter, single damage and multiple damages under noise-free and noisy conditions will be studied, respectively. For the single damage scenario, damage is introduced to the fifth and seventh element, separately. Multiple damages mean that damage is introduced to fifth and seventh element, simultaneously. Five scenarios are considered for single damage and multiple damages, respectively. Details for these damage scenarios are shown in Table 1. Including the intact scenario, 16 scenarios are taken into consideration. For noisy cases, noise is introduced to all the scenarios with 2% and 5% random noise, respectively. Note that different damaged scenarios are noted with ‘Intact’, ‘D1’, ‘D2’, …, ‘D5’, respectively.

### 3.2. Results Analysis

The proposed methodology for inspecting structural damage is calculated for the cantilever beam, and the results are drawn hereafter. In this section, the transmissibility is derived; unlike the previous investigations [10,11,12,13,14,15], transmissibility is selected upon a specified reference. This study uses ab average transmissibility with a changing reference for all available cases; this is the same in the further experiment investigation. The reason to use this averaged transmissibility is that this will avoid the reference selection, which would be easier for beginners. In this free-free numerical beam, the references change from node one to node 11, and then transmissibilities T(1, 1), T(2, 1), …, T(11, 1); T(1, 2), T(2, 2), …, T(11, 3); T(1, 3), T(2, 3), …, T(11, 3); …, T(1, 11), T(2, 11), …, T(11, 11). Then, by averaging all these references (node one to node 11), eleven averaged transmissibilities AT(1, :), AT(2, :), …, AT(11, :) would be derived. With these derived averaged transmissibilities, in light of all scenarios including undamaged and damaged ones, all damage-sensitive indicators would be obtained and applied for further predictions.

#### 3.2.1. Noisy Free Scenarios

**Figure 3:**
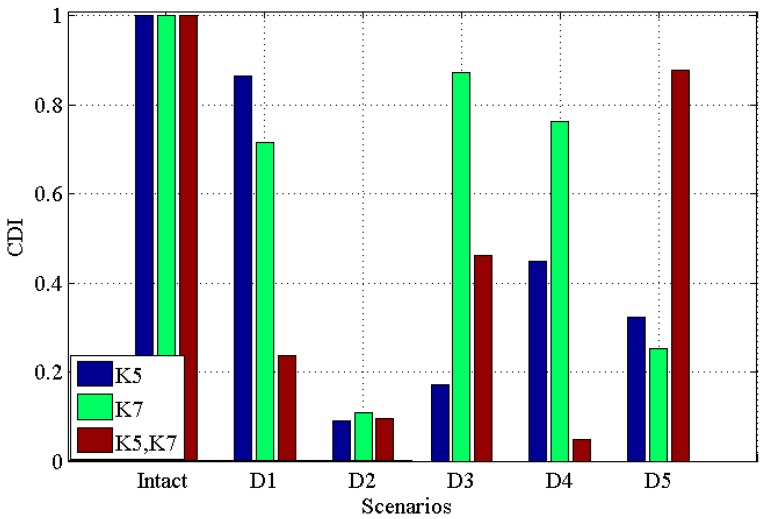
Correlation-based damage indicator (*CDI*) for five damaged scenarios with intact scenario for single damage in K5, single damage in K7, multiple damages in K5 and K7 respectively.

**Figure 4:**
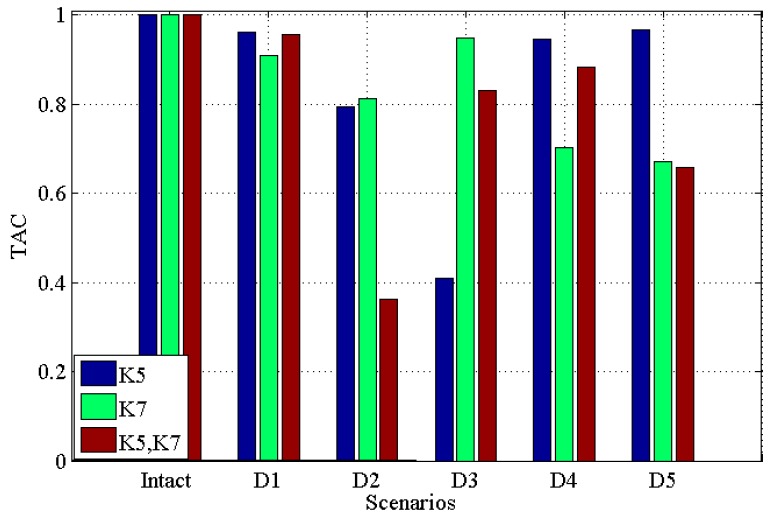
Transmissibility assurance criterion (TAC) for five damaged scenarios with intact scenario for single damage in K5, single damage in K7, multiple damages in K5 and K7, respectively.

Figure 3 and Figure 4 demonstrate the *CDI* and *TAC* for five damage scenarios along with the undamaged scenario for single damage in K5, single damage in K7, multiple damages in K5 and K7, respectively. Note that in the figure, ‘K5’ means single damage in K5 is introduced into the structure; ‘K7’ means single damage is introduced in K7 is introduced into the structure, while ‘K5, K7’ means the multiple damages introduced into K5 and K7 simultaneously. This will be the same in the latter figures.

From these two figures, it can be seen that (i) for both single and multiple damages, all five damaged scenarios are successfully identified from the intact scenario; (ii) for ‘D1’ to ‘D4’, *CDI* for multiple damages decrease more than single damage; (iii) generally, the *CDI*s for both single damage (in ‘K5’ and ‘K7’, respectively) and multiple damages (in ‘K5’ and ‘K7’) vary more than *TAC*, which may suggest that *CDI* has a better performance in discriminating the damaged scenarios from the intact scenario.

#### 3.2.2. Noisy Scenarios

**Figure 5:**
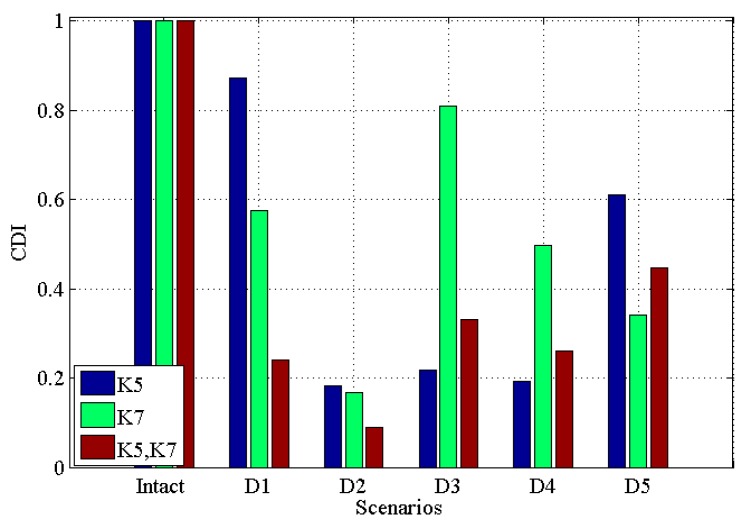
*CDI* for five damaged scenarios with intact scenario with 2% noise for single damage in K5, single damage in K7, multiple damages in K5 and K7, respectively.

**Figure 6:**
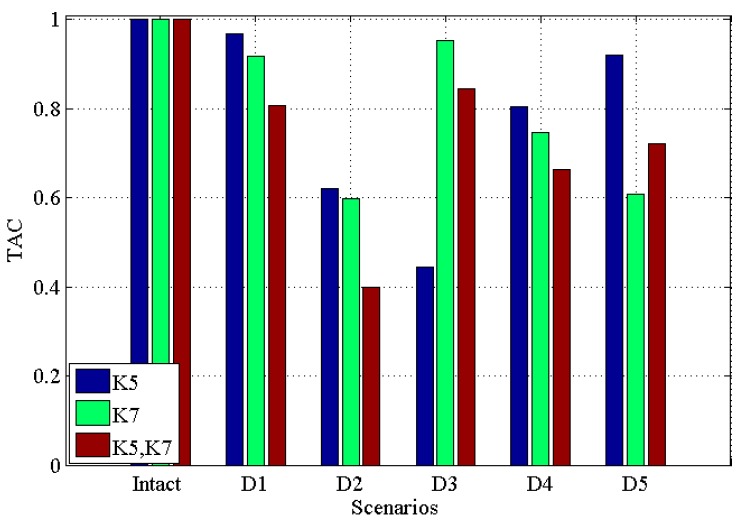
*TAC* for five damaged scenarios with intact scenario with 2% noise for single damage in K5, single damage in K7, multiple damages in K5 and K7, respectively.

Figure 5 and Figure 6 illustrate the results under the condition of 2% noise, *CDI* and *TAC* for single damage in K5, single damage in K7, multiple damages in K5 and K7 with 2% noise, respectively. From these two figures, one can see that (i) all damaged scenarios including single and multiple damages are successfully discriminated from the intact scenario; (ii) similar to the noisy free case, the *CDI* in Figure 5 varies more than TAC in Figure 6, which implies the potential better performance of *CDI* in determining damaged scenarios from intact one in comparison with TAC; (iii) generally, *CDI* and TAC for multiple damages vary more than that in single damage from the comparison between Figure 5 and Figure 6 with Figure 3 and Figure 4; (iv) comparing Figure 5 and Figure 6 with noisy free cases in Figure 3 and Figure 4, it can be found that 2% noise does not make the detection of damage challenging.

**Figure 7:**
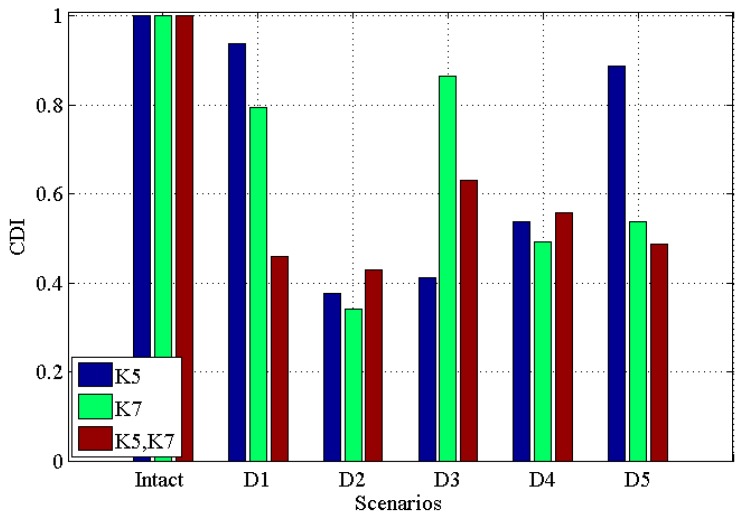
*CDI* for five damaged scenarios with intact scenario with 5% noise for single damage in K5, single damage in K7, multiple damages in K5 and K7, respectively.

**Figure 8:**
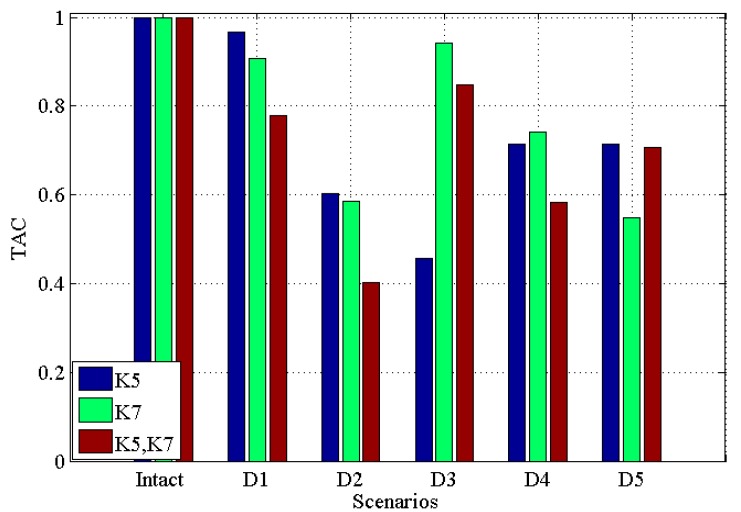
TAC for five damaged scenarios with intact scenario with 5% noise for single damage in K5, single damage in K7, multiple damages in K5 and K7, respectively.

Figure 7 and Figure 8 illustrate *CDI* and *TAC* under the condition with 5% noise for single damage in K5, single damage in K7, multiple damages in K5 and K7 with 5% noise, respectively. From these two figures one can see that: (i) all damaged scenarios including both single and multiple damages are successfully determined from the intact one, which proves the feasibility of the proposed indicators *CDI* and *TAC* to be effective in identifying damages; (ii) similar to the results obtained in previous noisy free case (Figure 3 and Figure 4) and 2% noisy case (Figure 5 and Figure 6), *CDI* varies more than *TAC* generally, which shows consistency with those results obtained in noisy free case (Figure 3 and Figure 4) and 2% noisy case (Figure 5 and Figure 6); (iii) for both *CDI* in Figure 7 and TAC in Figure 8, 5% noise does not make the proposed damage detection methodology vague, since the results obtained still demonstrate a clear difference between damaged scenarios and intact scenario in both *CDI* (Figure 7) and TAC (Figure 8).

## 4. Experimental Validation

### 4.1. Model Description

**Figure 9:**
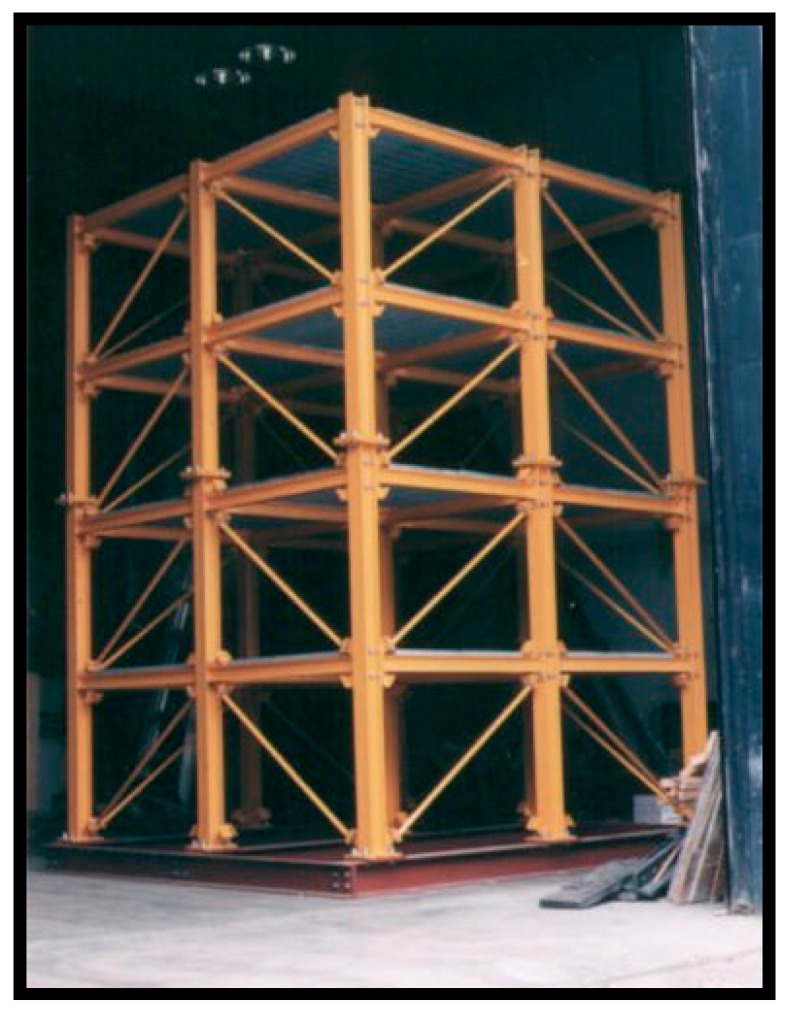
Model of the benchmark structure [40].

In order to further validate the proposed damage detection procedure, a four-story and two-bay by two-bay steel frame scale model structure shown in Figure 9 (American Society of Civil Engineers (ASCE) benchmark problem) [37] is employed. Detailed information for setting the experiment can be found in [38,39,40]. For the benchmark, several damage scenarios are modeled by adding additional mass, and changing braces or loosening bolts at the beam-column connections. The various damage scenarios are indicated in Table 2 [40]. The cause for employing this example for the validation of the proposed damage detection procedure is that this benchmark has been widely used in the literature and previous studies revealed its consistent performance. In this study, the experimental data obtained using a hammer in Phase II is used. In previous investigations, transmissibility has also proved to be applicable in this ASCE benchmark [8], where PCA has been applied in condensing the transmissibility; afterwards, the distance measures are used in discriminating the PCA condensed transmissibilities between damaged scenarios and undamaged ones, including city block distance, Chebyshev distance, Minkowski distance, Euclidean distance, and Mahalanobis distance.

### 4.2. Result Analysis

In this case, as the sensors are mounted at different floors and the directions are distinct, averaging all the transmissibilities for all the directions would not be a good option. The transmissibility is constructed after grouping into four parts: (1) first, fifth, ninth, thirteenth sensors; (2) second, sixth, tenth, fourth sensors; (3) third, seventh, eleventh, fifteenth sensors; (4) fourth, eighth, twelfth, sixteenth sensors. Then, transmissibilities T(1, :), T(5, :), T(9, :), T(13, :) with references at each sensor 5, 9 and 13; T(2, :), T(6, :), T(10, :), T(14, :) with references at each sensor 6, 10 and 14; and T(3, :), T(7, :), T(11, :), T(15, :) with references at each sensor 7, 11 and 15; T(4, :), T(8, :), T(12, :), T(16, :) with references at each sensor 8, 12 and 16. One may ask here why the separation is as above; the reason hidden behind this is that this truly depends on the engineers’ experience. To select a series of transmissibilities that sufficiently characterize the structural state would be sufficient.

**Figure 10:**
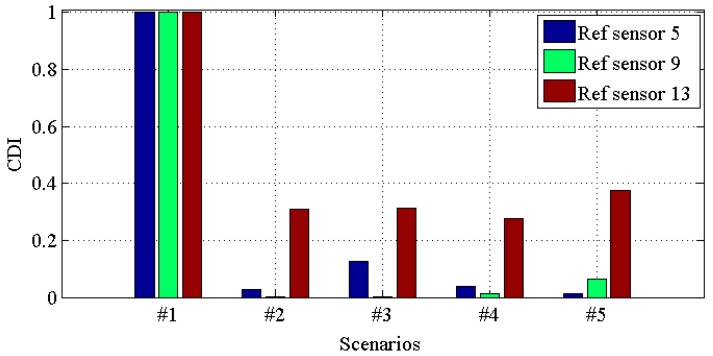
*CDI* for transmissibilities T(1, :), T(5, :), T(9, :) and T(13, :) with reference sensor 5, 9 and 13 for damage scenario #1 to #5.

**Figure 11:**
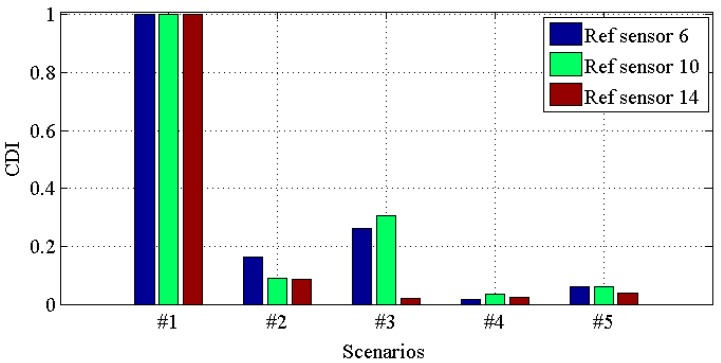
*CDI* for transmissibilities T(2, :), T(6, :), T(10, :) and T(14, :) with reference sensor 6, 10 and 14 for damage scenario #1 to #5.

**Figure 12:**
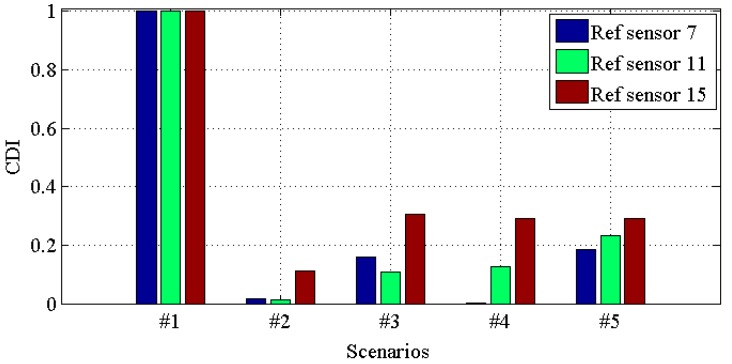
*CDI* for transmissibilities T(3, :), T(7, :), T(11, :) and T(15, :) with reference sensor 7, 11 and 15 for damage scenario #1 to #5.

**Figure 13:**
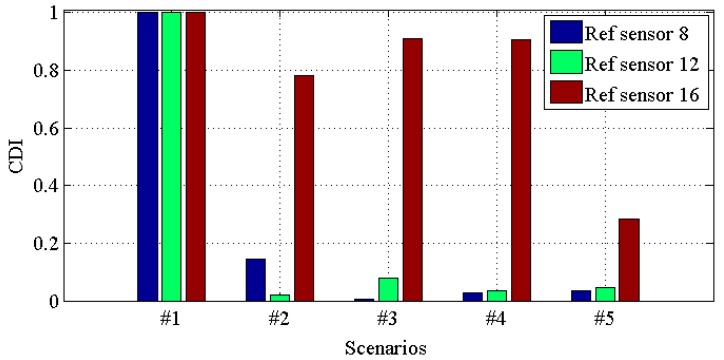
*CDI* for transmissibilities T(4, :), T(8, :), T(12, :) and T(16, :) with reference sensor 8, 12 and 16 for damage scenario #1 to #5.

Figure 10, Figure 11, Figure 12 and Figure 13 demonstrate the *CDI* of transmissibilities T(1, :), T(5, :), T(9, :), T(13, :) with reference sensor 5, 9 and 13; T(2, :), T(6, :), T(10, :), T(14, :) with reference sensor 6, 10 and 14; T(3, :), T(7, :), T(11, :), T(15, :) with reference sensor 7, 11 and 15; T(4, :), T(8, :), T(12, :), T(16, :) with reference sensor 8, 12 and 16; for scenario #1 to #5, respectively. From these four figures, one can conclude that: (i) all the damaged scenarios from #2 to #5 are successfully determined from the intact scenario #1; (ii) it is challenging to draw any insight of quantifying the different scenarios; (iii) *CDI* has different performances with different reference sensors, and this implies the importance of the reference selection. For instance, *CDI* for T(4, :), T(8, :), T(12, :) and T(16, :) with reference sensor 16 varies much less than *CDI* for others in Figure 10, Figure 11 and Figure 12.

**Figure 14:**
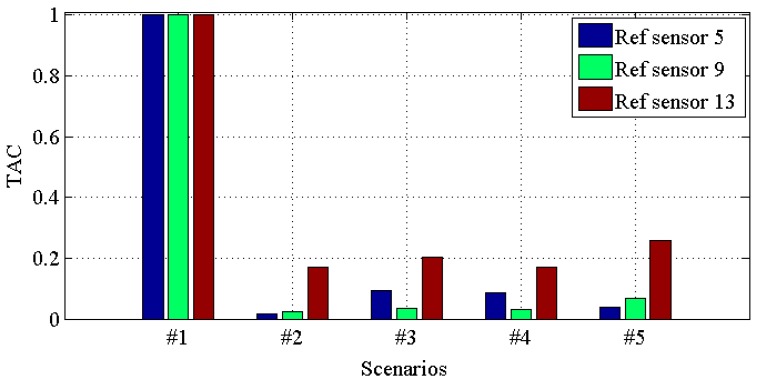
*TAC* for transmissibilities T(1, :), T(5, :), T(9, :) and T(13, :) with reference sensor 5, 9 and 13 for damage scenario #1 to #5.

**Figure 15:**
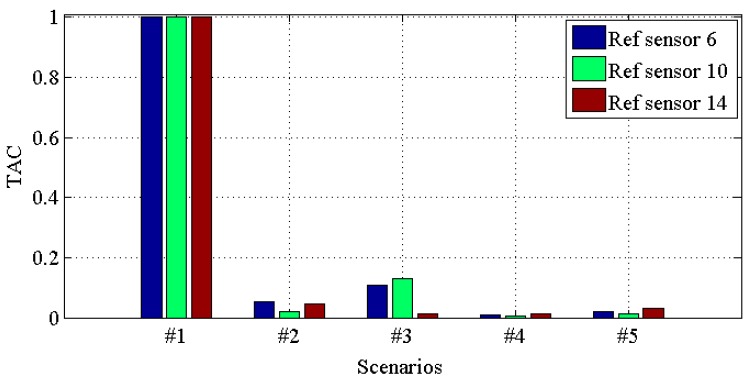
*TAC* for transmissibilities T(2, :), T(6, :), T(10, :) and T(14, :) with reference sensor 6, 10 and 14 for damage scenario #1 to #5.

**Figure 16:**
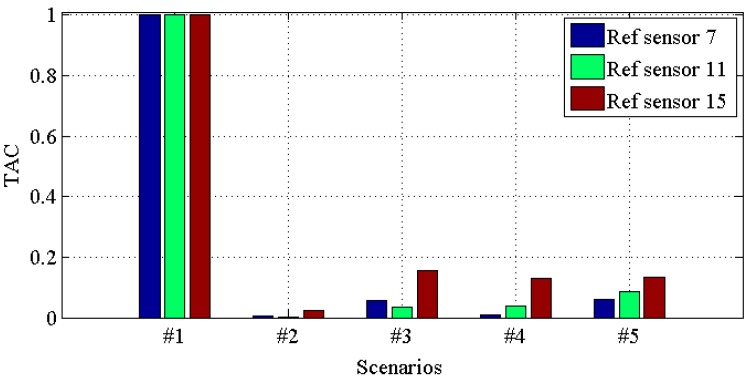
*TAC* for transmissibilities T(3, :), T(7, :), T(11, :) and T(15, :) with reference sensor 7, 11 and 15 for damage scenario #1 to #5.

**Figure 17:**
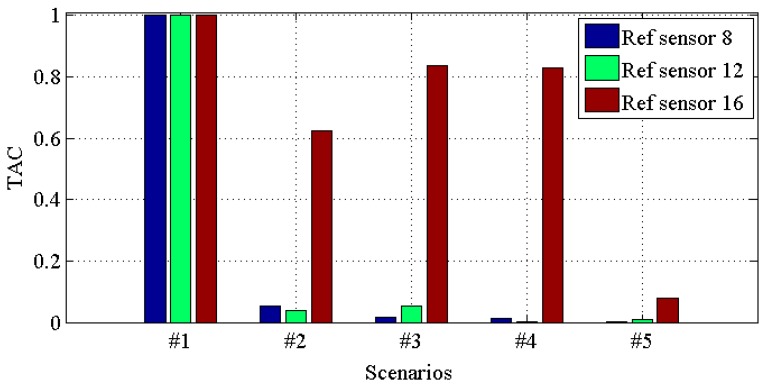
*TAC* for transmissibilities T(4, :), T(8, :), T(12, :) and T(16, :) with reference sensor 8, 12 and 16 for damage scenario #1 to #5.

Figure 14, Figure 15, Figure 16 and Figure 17 demonstrate *TACs* of transmissibilities T(1, :), T(5, :), T(9, :), T(13, :) with reference sensor 5, 9 and 13; T(2, :), T(6, :), T(10, :), T(14, :) with reference sensor 6, 10 and 14; T(3, :), T(7, :), T(11, :), T(15, :) with reference sensor 7, 11 and 15; T(4, :), T(8, :), T(12, :), T(16, :) with reference sensor 8, 12 and 16; for scenario #1 to #5, respectively. From these four figures, one can observe that: (i) similar to *CDI*s in Figure 10, Figure 11, Figure 12 and Figure 13, all the damaged scenarios from #2 to #5 are determined from the intact scenario #1 successfully; (ii) it is also arduous to draw out any damage quantification versus the different scenarios; (iii) similar to *CDI*s in Figure 10, Figure 11, Figure 12 and Figure 13, the reference setting also has effect in the damage detection results; which confirms the essence of reference.

## 5. Conclusions

This study first introduces the correlation coefficient into transmissibility and then constructs a sensitive feature, namely *CDI*, to characterize structural physical damage. This is also compared with the MAC indicator (*TAC*) in checking its applicability for damage detection. A numerical analysis and ASCE benchmark are conducted to unveil the feasibility of the proposed damage detection procedure; then, from the investigation illustrated above, some concluding remarks can be drawn out and summarized as follows:

(1) This study contributed an alternative criteria—*CDI*—in addition to the conventional damage sensitive indicators, and the correlation coefficient-based indicator *CDI* proved to be efficient and effective in detecting the structural damages, and it has a good tolerance to noise from the numerical analysis;
(2) The comparison between *CDI* and *TAC* proved that both features have a good capacity for identifying structural damages; namely, discriminating structural damages from the baseline—the undamaged scenario—while locating and quantifying the damages would be challenging for both of them;
(3) The proposed approach performs very well in distinguishing minor structural damage, which might be used in real-time SHM. Further investigation with more accurate experiment data is needed to achieve the potential for the locating and quantifying of damages;
(4) For a nonlinear problem such as hysteretic damping, complex geometry and so on, further investigation by extending the proposed approach, such as using further accurate measured damping sensitive data, would give a potential satisfactary solution;
(5) For damage type recognition, i.e., to determine the damage type such as corrosion and cracking, further investigation awaits to unveil the kernel and the potentail application of transmissibiltiy.

## Figures and Tables

**Table 1:** Damage scenarios for the noisy free condition.

Damage Scenario	Single Damage		Multiple Damages
	Stiffness Reduction K5	Stiffness Reduction K7	Stiffness Reduction K5 and K7
Intact	0	-	-
D1	2%	2%	2%
D2	5%	5%	5%
D3	10%	10%	10%
D4	15%	15%	15%
D5	20%	20%	20%

**Table 2:** Test scenario description [40].

Damage Scenario	Case Description
#1	All braces
#2	Missing all east side braces
#3	Case #2 + remove one brace on floor 1
#4	Case #3 + remove one brace on floor 3
#5	Case #4 + loosen one connection
